# Milk/dairy products consumption and gastric cancer: an update meta-analysis of epidemiological studies

**DOI:** 10.18632/oncotarget.23496

**Published:** 2017-12-15

**Authors:** Shuai Wang, Mi Zhou, Alin Ji, Dahong Zhang, Jinjing He

**Affiliations:** ^1^ Department of Urology, Zhejiang Provincial People's Hospital, People's Hospital of Hangzhou Medical College, Hangzhou 310014, Zhejiang Province, China; ^2^ Department of Ophthalmology, Zhejiang Provincial People's Hospital, People's Hospital of Hangzhou Medical College, Hangzhou 310014, Zhejiang Province, China

**Keywords:** dairy products, gastric cancer, meta-analysis, epidemiological studies

## Abstract

The relationship between dairy consumption and gastric cancer risk has not been well studied. We therefore performed a update meta-analysis to evaluate the relationship. Published cohort and case-control studies were identified via computer searches and reviewing the reference lists of the key articles. Random effects meta-analysis was used to pool effects from 5 cohort and 29 case-control studies. The odds ratio for the overall association between dairy consumption and gastric cancer was 1.20 (95%confidence interval: 1.04–1.39). The combined risk estimate was similar for population-based case-control studies (odds ratio = 1.27, 95%confidence interval: 1.00–1.61), but was reduced for hospital-based studies (odds ratio = 1.22; 95%confidence interval: 0.95–1.57) and cohort studies (odds ratio = 0.99; 95%confidence interval: 0.77–1.28). There was high heterogeneity in overall analyses. In the population-based subgroup analyses, the odds ratio was 0.96 (95%confidence interval: 0.69–1.34) when considering five studies assessing exposure two or more years before interview, and the association strengthened (odds ratio = 1.91, 95%confidence interval: 1.60–2.28) when dairy consumption was evaluated one year or less prior to interview. In conclusion, we found adverse effect of dairy consumption associated with gastric cancer.

## INTRODUCTION

Gastric cancer is the fifth most common cancer worldwide with 952,000 cases diagnosed in 2012 [1]. With its poor survival, it holds the second most common cause of cancer death worldwide after lung cancer [2]. It is estimated that 21,320 people (13,020 men and 8,300 women) will be diagnosed with and 10,540 people will die of cancer of the stomach in 2012 in the United states [3]. Surgery is the most frequent treatment option for stomach cancers but prognosis is generally rather poor, with cohort and period estimate of 5-year relative survival rate below 25% [4]. Therefore, early intervention on modifiable risk factors of gastric cancer is very important. Given the ten-fold variation in disease incidence between population at the highest and lowest risk [2], dietary factors have been suggested to play a key role in the aetiology of the disease. The identification of foods and nutrients associated with gastric cancer could give an opportunity for prevention.

With regard to dietary factors, dairy products are important source of several nutrients, including animal fat, lactose, vitamins, calcium, and total energies. The Dietary Guidelines for Americans 2010 continue to recommend three daily serving of dairy products. Dairy products is such a common exposure that any small effect can result in a large population impact. Reviews and meta-analyses of available studies showed a 16% reduction bladder cancer risk in milk consumers [5], no effect on ovarian cancer risk [6], and a 11% increased in prostate cancer risk [7]. Investigation of gastric cancer has been less extensive. Many epidemiologic studies that examined the association diary product consumption and risk of gastric cancer have produced conflicting results, with both inverse and positive associations reported. A previous pooling analysis by Huang et al. [8] supported the hypothesis that dairy products consumption decreased the risk of gastric cancer. However, the included eight case-control studies were all conducted in China, where the dairy products consumption is less common than in Western countries. Recently, another three meta-analyses of the correlation between dairy consumption and gastric cancer risk were performed. However, they appeared to be flawed because of confusion in the selection criteria and pooling of no adjusted risk estimates [9–11]. Thus, we aimed to update and quantitatively reassess the association between dairy products consumption and gastric cancer.

## RESULTS

We identified 41 case-control studies and five independent cohort studies that examined the risk of gastric cancer with milk/dairy consumption. Upon closer examination, 12 case-control articles did not provide a summary OR or its 95% CIs [12–23]. Three articles [24–26] was excluded because milk intake had been treated as a continuous variable, not discrete categories. One study [14] was updated by Palli et al [27]. Two cohort study provided hazard ratios [28, 29] instead of OR/RR. The remaining 29 case-control studies and 5 cohort studies are presented in Supplementary Table 1. They were carried out in 15 countries and areas. 7 were conducted in the United States [30–36], 8 were in Europe [27, 37–43], and 14 were in Asia [44–57] , 3 in Latin America [58–60], and 2 in Turkey [61, 62]. 5 cohort studies [32, 35, 36, 38, 53] regarding the association between dairy consumption and risk of gastric cancer were identified. The characteristics of these studies were also presented in Supplementary Table 1. 25 studies used only histologically confirmed cases, two stated that respectively 98% [30] and 90.2% [42] of cases had histological confirmation, five studies only indicated having used cancer registries to identify cases [34–36, 38, 53], and two declared the cases were newly diagnosed at certain hospitals [61, 62].

The number of cases enrolled in these case-control studies ranged from 41 to 1111, with a sum of 8956. And corresponding numbers of controls ranged from 128 to 36490, with a sum of 78319. 13 case-control studies selected controls from populations and the other 17 studies from hospitals. Hoshiyama et al. [44]. used both hospital and population controls for comparison separately. Among the 34 studies, 20 reported on dairy food intake and 21 reported on milk intake. Gastric cancer risk was reported separately by both dairy and different types of milk in several studies [31, 38–41, 56, 60], as shown in Supplementary Table 1. Among the 29 case-control studies, dietary information was assessed for the period at least 2 years before onset of symptoms or diagnosis in 9 studies [27, 31, 37, 39, 42, 43, 55, 57, 59], while one years or less prior to interview or diagnosis in 9 studies [30, 40, 41, 45, 49, 50, 56, 60, 61], and the remaining 11 studies did not mention the exposure period.

**Figure 1:**
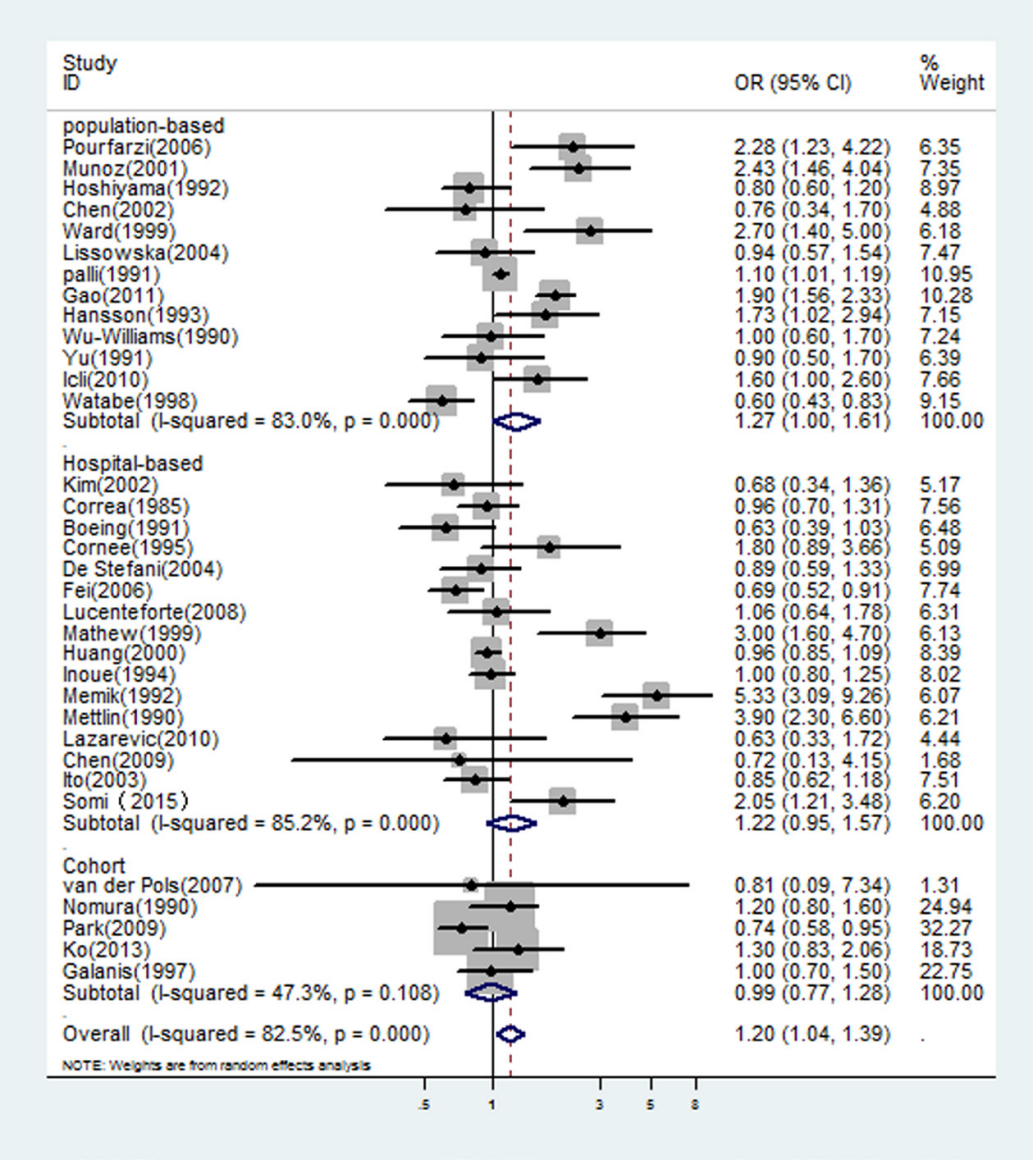
In overall studies, risk estimates of dairy consumption associated with gastric cancer

The multivariable-adjusted ORs for each study and all studies combined for the highest versus lowest categories of dairy consumption in relation to gastric cancer risk are shown in Figure 1. A significant positive association was seen between dairy consumption and gastric cancer risk: the pooled OR was 1.20 (95% CI: 1.04–1.39) in a random-effects analysis. There was a statistically significant heterogeneity across studies (I^2^ = 83.0%). The combined risk estimate was 1.27 (95% CI: 1.00–1.61) for population-based case-control studies and 1.22 (95% CI: 0.95–1.57) for hospital-based case-control studies, with statistically significant heterogeneity (see Figure 1). In Table 1, we performed subgroup analyses based on study design, geographical region (Europe, Asia, USA, Latin America), and exposure period (at least 2 year prior to interview, one years or less prior, and not specified). The OR estimates showed dairy consumption was consistently associated with an increased risk of gastric cancer, although some of the results were nonsignificant and with significant heterogeneity.

**Table 1:** Summary of pooled odds ratio of gastric cancer for the highest vs. the lowest level of dairy intake by study design, geographical region, exposure period, and specific dairy products

Subgroup		Number of studies	Poole RR (95% CI)	*Q*-test for heterogeneity *P*value (I^2^ score)
Study design				
Case-control	Hospital-based	16	1.22 (0.95–1.57)	I^2^ = 85.2% *P* = 0.000
Population-based	13	1.27 (1.00–1.61)	I^2^ = 83.0% *P* = 0.000	
Cohort		5	0.99 (0.77–1.28)	I^2^ = 47.3% *P* = 0.108
Geographical region				
	Europe	8	1.05 (0.84–1.32)	I^2^ = 41.3% *P* = 0.103
	Asia	14	1.11 (0.88–1.40)	I^2^ = 85.4% *P* = 0.000
	USA	7	1.06 (0.77–1.46)	I^2^ = 79.4% *P* = 0.000
	Latin America	3	1.76 (0.83–3.73)	I^2^ = 84.7% *P* = 0.001
Exposure period				
	≥ 2 years prior to interview	9	0.99 (0.78–1.26)	I^2^ = 71.0% *P* = 0.001
	≤ 1 years prior to interview	8	1.35 (1.02–1.80)	I^2^ = 84.5% *P* = 0.000
	not specified	12	1.41 (0.93–2.14)	I^2^ = 89.1% *P* = 0.000
Specific dairy products				
	Milk	21	1.44 (1.15–1.81)	I^2^ = 82.7% *P* = 0.000
	Dairy foods	20	1.08 (0.90–1.30)	I^2^ = 78.7% *P* = 0.000
	Cheese	5	1.22 (0.76–1.95)	I^2^ = 77.0% *P* = 0.000

**Figure 2:**
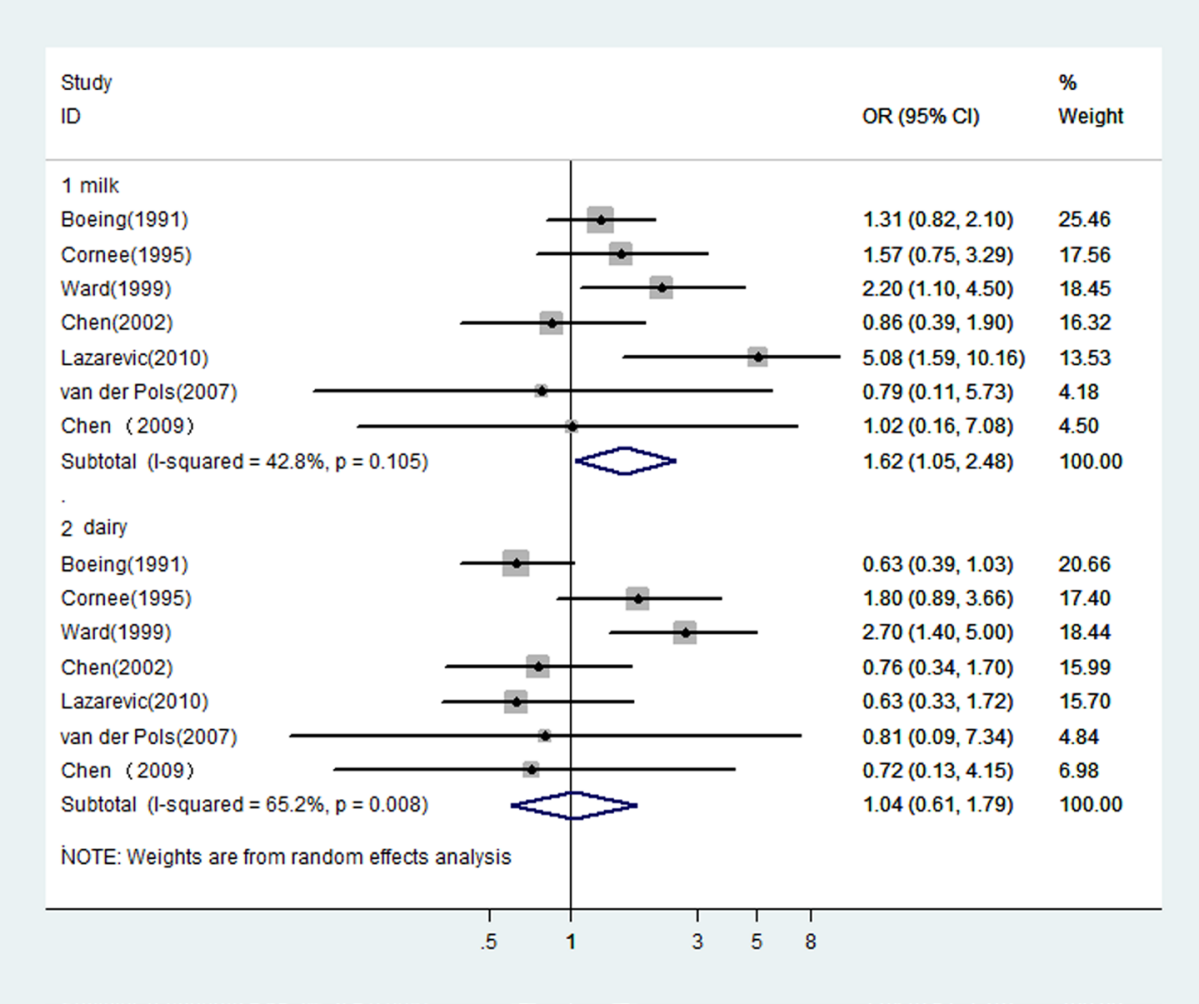
In those studies reported dairy and milk separately, risk estimates of gastric cancer associated with milk or dairy foods

In fact, it was difficult to classify these dairy studies because of the ambiguous or different definitions of food items in each questionnaire. In general, there were items of milk and dairy products, which were used in the included studies. According to this classification, we observed a significant relation of milk intake with an increased risk of gastric cancer (OR = 1.44, 95% CI: 1.15–1.81), while the relation of dairy foods intake was not significant (OR = 1.08, 95% CI: 0.90–1.30). When pooled estimates from the seven studies [31, 38–41, 56, 60] where reported dairy foods and milk separately, the similar results were observed (OR = 1.62, 95% CI: 1.05–2.48 for milk; OR = 1.04, 95% CI: 0.61–1.79 for dairy foods, see Figure 2).

**Figure 3:**
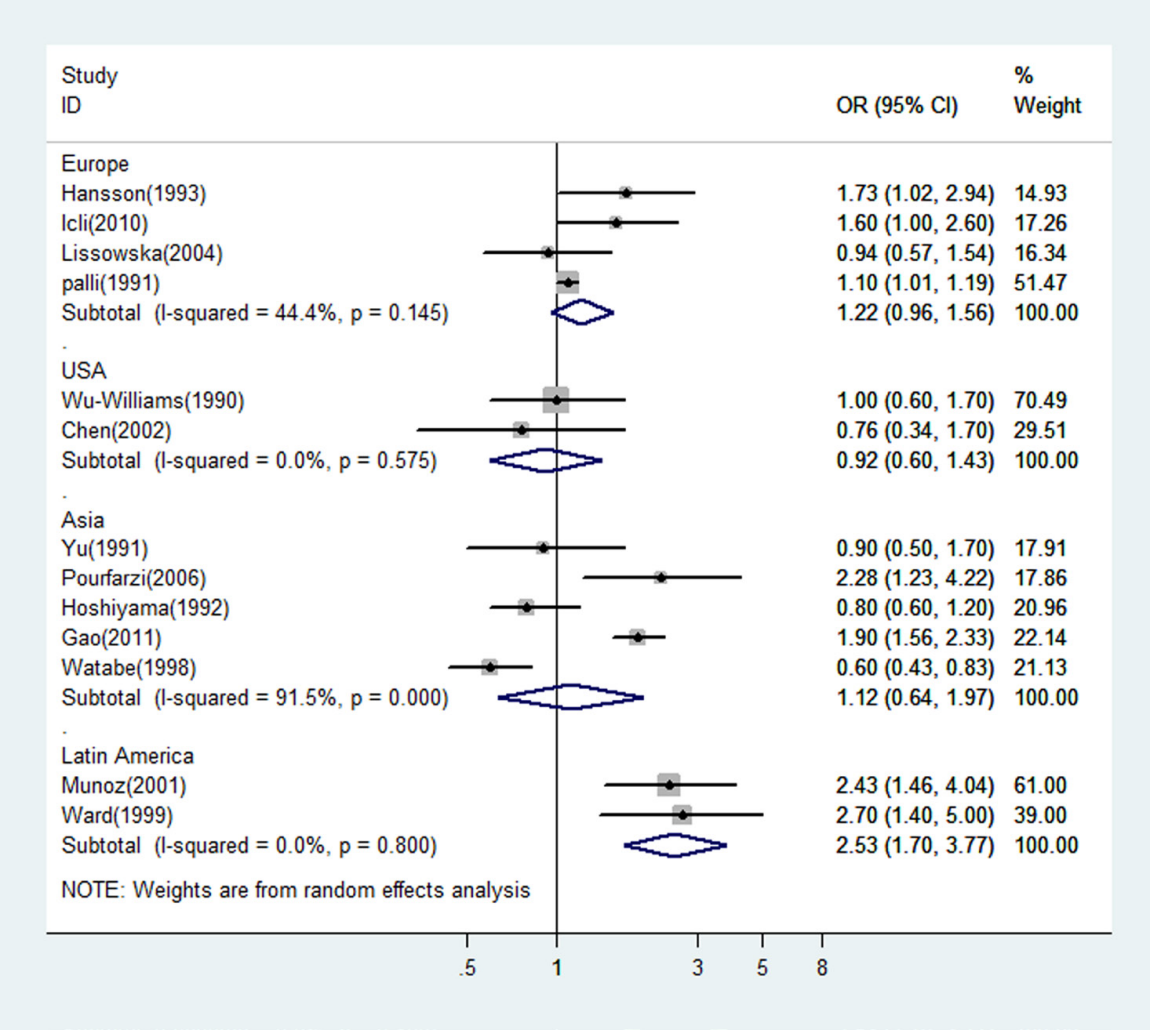
A forest plot showing risk estimates from population-based case-control studies, estimating the association between gastric cancer and dairy consumption by different geographical regions

**Figure 4:**
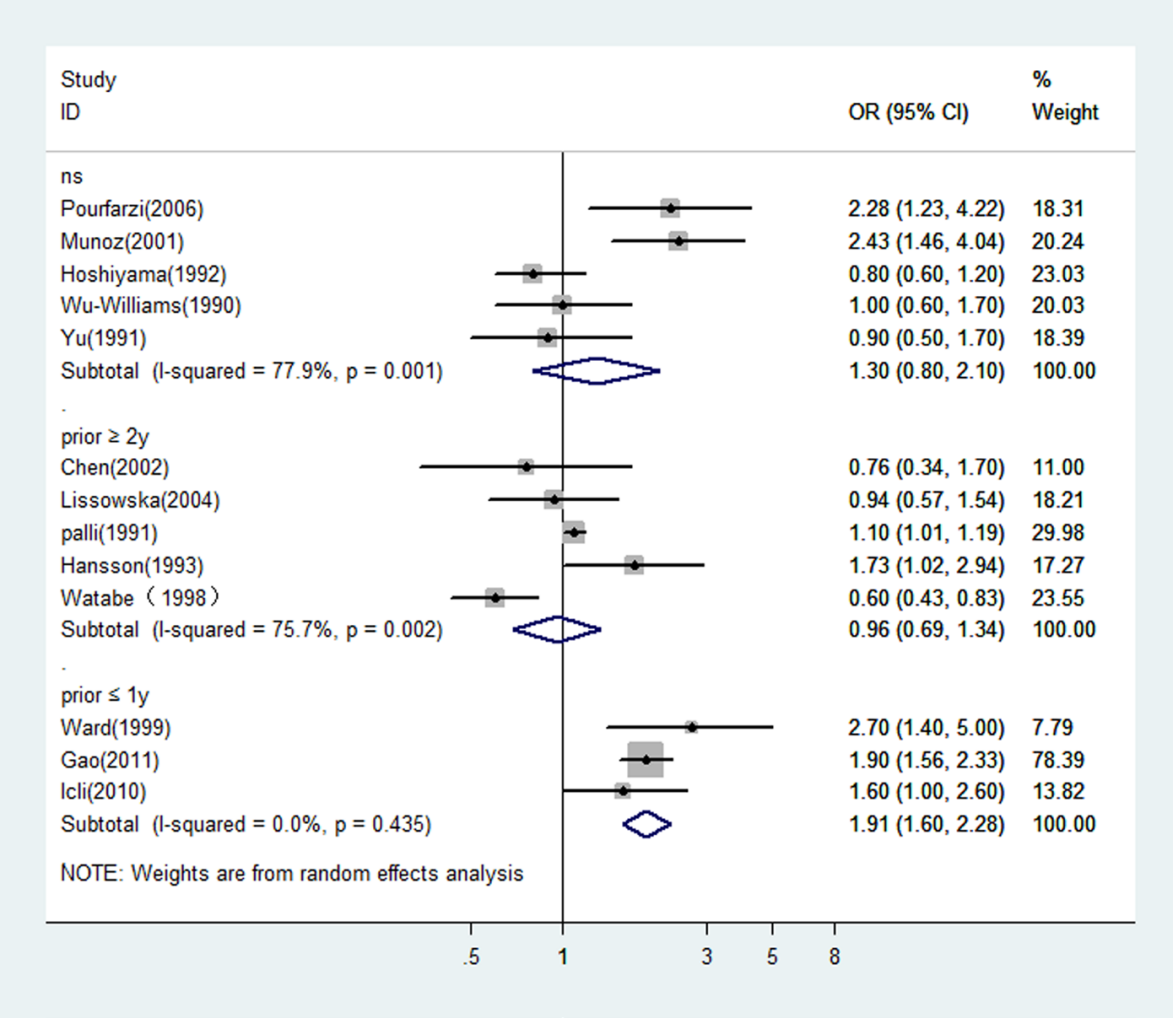
In population-based case-control studies, risk estimates of gastric cancer associated with different dairy consumption exposure period NS: not specified; prior ≤ 1 y: evaluated dairy consumption one year or less prior to the interview; prior ≥ 2 y: evaluated dairy consumption two or more years prior to the interview.

Since the type of control (hospital or population) would be a potential source of heterogeneity for meta-analysis and the design with population control has a great advantage over that with hospital control, we estimated the OR of dairy consumption limited in the studies with population controls. The OR was 1.12 (95% CI: 0.64–1.97 *P* = 0.0000) for the five Asian studies, and 1.22 (95% CI: 0.96–1.56 *P* = 0.145) for the four studies from Europe. There was statistically significant heterogeneity among the Asian studies, but not among the European studies (see Figure 3). Figure 4 showed the risk estimates of gastric cancer associated with different dairy consumption exposure period in population-based case-control studies. The combined odds ratio was 0.96 (95% CI: 0.69–1.34, *P* = 0.002) for those assessing exposure two or more years before interview, and 1.91 (95% CI: 1.60–2.28, *P* = 0.435) when dairy consumption was evaluated closer to the time of interview. And the pooled OR from the remaining studies which did not mention the exposure period was 1.30 (95% CI: 0.80–2.10, *P* = 0.001).

Sensitivity analyses by sequentially excluding one study in each turn to examine the influence of a single study on the overall estimate suggest the overall risk estimates did not substantially modified by any single study (data not show). The funnel plot showed some asymmetry among overall studies, while both the Begg's test (*P* = 0.064) and the Egger's test (*P* = 0.290) for publication bias were nonsignificant. Among the population-based case-control studies, there was no evidence of significant publication bias either with the Egger's test (*P* = 0.272) or Begg's test (*P* = 0.528).

## DISCUSSION

Results of our meta-analysis support a positive association between dairy consumption and the risk of gastric cancer. There were substantial heterogeneity across studies of the associations of dairy consumption with gastric cancer risk. This is not surprising given the variation in definitions of food items in each questionnaire, characteristics of populations between studies, and analytic methodology. We conducted a meta-regression analysis to assess the effect of publication year, control type, sample size, geographical region, and exposure period on the heterogeneity. However, none was identified as a possible source of heterogeneity among all the included studies or among population-based case-control studies (*P* > 0.05 for all). As indicated by our subgroup analyses, the type of control combined with exposure period likely contributed to the observed heterogeneity.

In 11 studies with a positive association between dairy products consumption and gastric cancer, five studies attributed it to that case may drink more milk to control symptoms of the disease such as dyspepsia, four studies did not investigate the reason, one study thought it may due to design and type of the studies, sample size, genetic factors and different food habits, one study considered it may relate to inflammation due to lactose intolerance, or carcinogen contamination of the milk. The components in dairy products are complex. It is of interest to note that dairy products have some components that could potentially increase risk of certain cancer and other components could decrease it. For example, insulin-like growth factor (IGF-I) concentrations in cows’ milk have been reported to range from 6 to 162 ng/ml [63]. Insulin-like growth factor type 1 receptor (IGF-IR) have been found expressing in surgical GC specimens [64]. IGF-IR is a cell membrane receptor that is activated by its ligands, IGF-I and IGF-II, then participates in cell proliferation, differentiation, prevention of apoptosis, and malignant transformation. However, additional studies need to be performed to provide evidence that IGF-I in milk has activity in the stomach where releases hydrochloric acid and remain highly acidic. In addition, several carcinogen in feedstuffs consumed by dairy animals could pass into milk. The International Agency for Research on Cancer (IARC) classified bracken fern (BF) in Group 2B, as possibly carcinogenic to humans. It has been discovered that ptaquiloside (Pta), which has been demonstrated to be the major carcinogen of BF, is indeed passed into milk [65]. Epidemiological studies reported a higher prevalence of gastric cancer in bracken fern-infested areas of northern Wales [13] and western Venezuela [66]. In Wales, milk has been postulated as a vector for carcinogenic compounds. Meanwhile, it is a worldwide problem that cows’ milk has been polluted by pesticides. In animal studies, many pesticides have been shown to be carcinogenic or tumor promoters. So, it is very important to ensure the dairy products with high hygienic standards.

On the other hand, estrogen in milk, which may contribute to the etiology of prostate cancer [67], may have a protective effect on gastric cancer. Recently, a nested case-control study within a prospective cohort, and meta-analysis demonstrated that the use of menopausal hormone therapy had a reduced risk of gastric cancer [68]. Besides, several components in dairy products, including vitamin D, calcium, conjugated linoleic acids, may be responsible for a protective association between dairy and gastric cancer.

Our study had several limitations that affected interpretation of the results. First, milk and dairy products are a collection of several products, the ambiguous or differing definitions of dairy items in each questionnaire may result in inaccurate estimates. In addition, measurement units for dairy intake varied and intake levels ranged widely across the studies included in our meta-analysis. For example, the lowest intake categories ranged from 0 to 89 g/d for dairy products.

Second, only English-language articles that had been published were included. We did not attempt to uncover unpublished observations and did not include studies with insufficient information to estimate an adjusted OR, which could bring publication bias, even though the Egger's test and Begg's test yielded the same conclusions without evidence of any potentially missed unpublished studies.

Third, in case-control studies, dairy food consumption among controls may not represent the target population, and bias is even more probable with hospital controls. Symptoms, such as dyspepsia, might impel cases to drink more milk. This differential information bias could lead to a misleading adverse effect. The results of stratified analyses by the exposure period seem support this hypothesis. The association was significant positive when combined the studies assessing exposure one year or less prior to the interview, and turned to marginally significant when pooled the studies that dairy consumption was evaluated two or more years before interview. So it seems beneficial to screen by gastroscopy for the high-risk patients, especially for the crowd who recently increase the milk consumption. Observational cohort studies, in which the evaluation of diet is unaffected by symptoms, should ideally provide much more reliable evidence. The five cohort studies identified in this analysis showed neither a positive nor inverse association between dairy consumption and gastric cancer risk.

## MATERIALS AND METHODS

### Search strategy

We identified relevant publications in the MEDLINE database using PubMed, Web of Science, and the Cochrane Library up to March 2016. The searches were limited to studies published in English. Search terms included ‘‘gastric cancer,’’ ‘‘stomach cancer,’’ or ‘‘stomach neoplasm’’ combined with ‘‘dairy product,’’ ‘‘milk,’’ or ‘‘diet”. The titles and abstracts were scanned to exclude any clearly irrelevant studies. The full texts of the remaining articles were read to determine whether they contained information on the topic of interest. In order not to omit relevant articles, the reference lists provided by the identified papers was additionally hand-searched.

### Search selection

**Figure 5:**
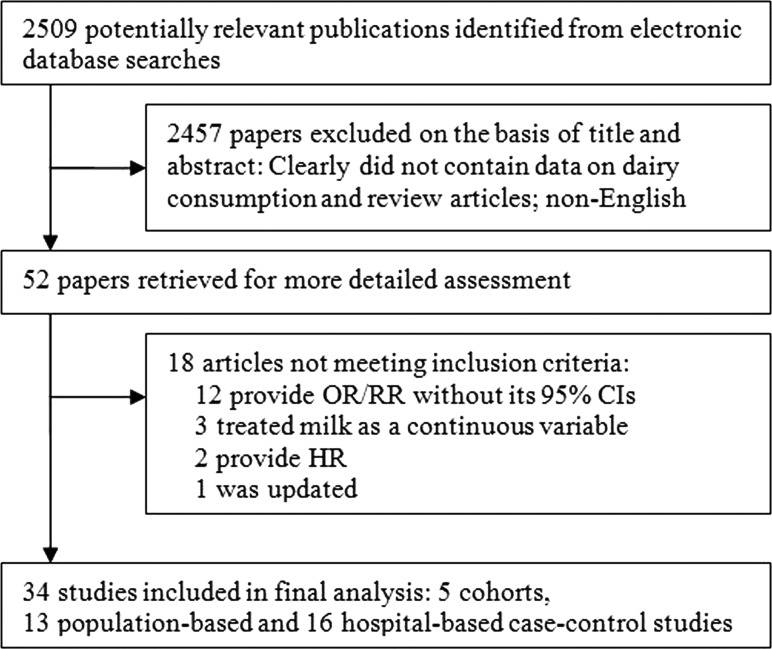
Flowchart of study selection process HR, hazard ratios; OR, odds ratio; RR, relative risk; CIs, confidence intervals.

To be included in our meta-analysis, eligible studies had to fulfill all of the following inclusion criteria: 1) case–control or cohort study published as an original article, 2) papers reported in English between 1980 and March 2016, 3) the outcome of interest was gastric cancer incidence, 4) odds ratio (OR) or relative risk (RR) with corresponding 95% confidence intervals (CIs) for the highest versus lowest categories of dairy consumption were reported, and 5) adjustment made for potential risks. We excluded studies that reported date on mortality from gastric cancer. In studies with overlapping patients or controls, only the latest or the most informative were included. Any study with inconsistent or erroneous data was excluded. Meeting abstracts with insufficient data or unpublished reports were not considered (Figure 5).

### Data extraction

Information from the included studies was extracted independently by two researchers (J.H. and S.W.). Conflicting evaluations were resolved by discussion. If a consensus still could not be reached, the senior investigator (D.Z.) made the final decision.

We extracted the first author's name, the year of publication, country in which the study was conducted, the study design, study period, sample size, mean age or age range of study subjects, dairy products consumption levels, the dairy product items, adjusted covariates, the method of assessment of dairy product intake, and exposure period. Quality assessment was performed based on the Newcastle-Ottawa Quality Assessment Scale, which is a validated scale for nonrandomized studies in meta-analyses. Considering the rare incidence of gastric cancer, the RR was assumed approximately the same as OR, and we report all results as OR for simplicity. Because the dairy product consumption categories varied between studies, we chose the highest and lowest levels of dairy product consumption categorized in each of the studies. Definitions of the levels used in each of the studies are shown in the ‘‘dairy product consumption levels’’ column of Supplementary Table 1. Our main analyses were focused on the associations between consumption of dairy and risk of gastric cancer. We used the reported OR for dairy intake when were provided. When gastric cancer risk was reported separately by several dairy items (e.g., milk or cheese), whole milk or milk was chosen because it is more widely consumed in the world than other items and contains all dairy substances and thus reflects the true nature of dairy. If both hospital and population controls were used for comparison separately, the result of the population control was chosen for the analysis. When a study provided more than one estimate, we selected the most completely adjusted estimate, and when results were available according to gender or ethnicity we included all estimates in the final analysis as if obtained from different studies.

### Statistical analysis

The data from individual studies were pooled by use of the random-effect model with the DerSimonian-Laird method [69], which considers within-study and between-study variation. We performed subgroup analyses based on different kinds of dairy products (milk and cheese). Certain items, such as yogurt, butter, or ice cream were seldom assessed in individual reports, these analyses were not performed. Additional subgroup analyses were also carried out to examine the effects of the study design, geographic region, and exposure period. Sensitivity analyses were conducted by omitting one study in each turn to investigate the influence of a single study on the overall risk estimate. The *Q*-statistic and I^2^ score were used to assess the between-study heterogeneity of results [70, 71]. Meta-regression analysis was used to assess the heterogeneity in publication year, study design, sample size, geographical region, and exposure period. Publication bias assessment was done using the Egger regression asymmetry test [72] and the Begg adjusted rank correlation test [73]. The statistical software used was Stata/SE 11.0 (Stata Corporation, College Station, TX), and the significance level was set to *P* < 0.05.

## CONCLUSIONS

In conclusion, we found a positive association between dairy consumption and gastric cancer risk from current published case-control studies using meta-analysis. This association strengthened when dairy consumption exposure was assessed closer to the time of interview or diagnosis. Meanwhile, the five cohort studies showed either a positive or inverse association between dairy consumption and gastric cancer risk. This suggests that the relationship between dairy consumption and gastric cancer may be partly affected by study design. Therefore, further large prospective studies are warranted to confirm these findings and further efforts should be made to elucidate the underlying mechanism.

## SUPPLEMENTARY MATERIALS TABLE





## References

[1] Jemal A, Bray F, Center MM, Ferlay J, Ward E, Forman D (2011). Global cancer statistics. CA Cancer J Clin.

[2] Brenner H, Rothenbacher D, Arndt V (2009). Epidemiology of stomach cancer. Methods Mol Biol.

[3] Siegel R, Naishadham D, Jemal A (2012). Cancer statistics, 2012. CA Cancer J Clin.

[4] Brenner H (2002). Long-term survival rates of cancer patients achieved by the end of the 20th century: a period analysis. Lancet.

[5] Mao QQ, Dai Y, Lin YW, Qin J, Xie LP, Zheng XY (2011). Milk consumption and bladder cancer risk: a meta-analysis of published epidemiological studies. Nutr Cancer.

[6] Qin LQ, Xu JY, Wang PY, Hashi A, Hoshi K, Sato A (2005). Milk/dairy products consumption, galactose metabolism and ovarian cancer: meta-analysis of epidemiological studies. Eur J Cancer Prev.

[7] Gao X, LaValley MP, Tucker KL (2005). Prospective studies of dairy product and calcium intakes and prostate cancer risk: a meta-analysis. J Natl Cancer Inst.

[8] Huang YX, Qin LQ, Wang PY (2009). [Meta-analysis of the relationship between dairy product consumption and gastric cancer]. [Article in Chinese]. Zhonghua Yu Fang Yi Xue Za Zhi.

[9] Guo Y, Shan Z, Ren H, Chen W (2015). Dairy consumption and gastric cancer risk: a meta-analysis of epidemiological studies. Nutr Cancer.

[10] Sun Y, Lin LJ, Sang LX, Dai C, Jiang M, Zheng CQ (2014). Dairy product consumption and gastric cancer risk: a meta-analysis. World J Gastroenterol.

[11] Tian SB, Yu JC, Kang WM, Ma ZQ, Ye X, Cao ZJ (2014). Association between dairy intake and gastric cancer: a meta-analysis of observational studies. PLoS One.

[12] Lee HH, Wu HY, Chuang YC, Chang AS, Chao HH, Chen KY, Chen HK, Lai GM, Huang HH, Chen CJ (1990). Epidemiologic characteristics and multiple risk factors of stomach cancer in Taiwan. Anticancer Res.

[13] Galpin OP, Whitaker CJ, Whitaker R, Kassab JY (1990). Gastric cancer in Gwynedd. Possible links with bracken. Br J Cancer.

[14] Buiatti E, Palli D, Decarli A, Amadori D, Avellini C, Bianchi S, Biserni R, Cipriani F, Cocco P, Giacosa A, Marubini E, Puntoni R, Vindigni C (1989). A case-control study of gastric cancer and diet in Italy. Int J Cancer.

[15] Tuyns AJ, Kaaks R, Haelterman M, Riboli E (1992). Diet and gastric cancer. A case-control study in Belgium. Int J Cancer.

[16] Gonzalez CA, Sanz JM, Marcos G, Pita S, Brullet E, Saigi E, Badia A, Riboli E (1991). Dietary factors and stomach cancer in Spain: a multi-centre case-control study. Int J Cancer.

[17] Ramon JM, Serra L, Cerdo C, Oromi J (1993). Dietary factors and gastric cancer risk. A case-control study in Spain. Cancer.

[18] Ursin G, Bjelke E, Heuch I, Vollset SE (1990). Milk consumption and cancer incidence: a Norwegian prospective study. Br J Cancer.

[19] La Vecchia C, Negri E, Decarli A, D’Avanzo B, Franceschi S (1987). A case-control study of diet and gastric cancer in northern Italy. Int J Cancer.

[20] Haenszel W, Kurihara M, Segi M, Lee RK (1972). Stomach cancer among Japanese in Hawaii. J Natl Cancer Inst.

[21] Kono S, Ikeda M, Tokudome S, Kuratsune M (1988). A case-control study of gastric cancer and diet in northern Kyushu, Japan. Jpn J Cancer Res.

[22] Xibin S, Moller H, Evans HS, Dixing D, Wenjie D, Jianbang L (2002). Residential Environment, Diet and Risk of Stomach Cancer: a Case-control Study in Linzhou, China. Asian Pac J Cancer Prev.

[23] Amadori D, Nanni O, Ricci M, Falcini F, Decarli A, Palli D, Buiatti E (1995). Hospital versus population controls in a retrospective study on diet and stomach cancer. Eur J Public Health.

[24] Navarro Silvera SA, Mayne ST, Risch H, Gammon MD, Vaughan TL, Chow WH, Dubrow R, Schoenberg JB, Stanford JL, West AB, Rotterdam H, Blot WJ, Fraumeni JF (2008). Food group intake and risk of subtypes of esophageal and gastric cancer. Int J Cancer.

[25] Pakseresht M, Forman D, Malekzadeh R, Yazdanbod A, West RM, Greenwood DC, Crabtree JE, Cade JE (2011). Dietary habits and gastric cancer risk in north-west Iran. Cancer Causes Control.

[26] De Stefani E, Deneo-Pellegrini H, Boffetta P, Ronco AL, Aune D, Acosta G, Mendilaharsu M, Brennan P, Ferro G (2009). Dietary patterns and risk of cancer: a factor analysis in Uruguay. Int J Cancer.

[27] Palli D, Bianchi S, Decarli A, Cipriani F, Avellini C, Cocco P, Falcini F, Puntoni R, Russo A, Vindigni C, Fraumeni JF, Blot BJ, Buiatii E (1992). A case-control study of cancers of the gastric cardia in Italy. Br J Cancer.

[28] Buckland G, Agudo A, Lujan L, Jakszyn P, Bueno-de-Mesquita HB, Palli D, Boeing H, Carneiro F, Krogh V, Sacerdote C, Tumino R, Panico S, Nesi G (2010). Adherence to a Mediterranean diet and risk of gastric adenocarcinoma within the European Prospective Investigation into Cancer and Nutrition (EPIC) cohort study. Am J Clin Nutr.

[29] Pham TM, Fujino Y, Kikuchi S, Tamakoshi A, Matsuda S, Yoshimura T (2010). Dietary patterns and risk of stomach cancer mortality: the Japan collaborative cohort study. Ann Epidemiol.

[30] Correa P, Fontham E, Pickle LW, Chen V, Lin YP, Haenszel W (1985). Dietary determinants of gastric cancer in south Louisiana inhabitants. J Natl Cancer Inst.

[31] Chen H, Ward MH, Graubard BI, Heineman EF, Markin RM, Potischman NA, Russell RM, Weisenburger DD, Tucker KL (2002). Dietary patterns and adenocarcinoma of the esophagus and distal stomach. Am J Clin Nutr.

[32] Nomura A, Grove JS, Stemmermann GN, Severson RK (1990). A prospective study of stomach cancer and its relation to diet, cigarettes, and alcohol consumption. Cancer Res.

[33] Wu-Williams AH, Yu MC, Mack TM (1990). Life-style, workplace, and stomach cancer by subsite in young men of Los Angeles County. Cancer Res.

[34] Mettlin CJ, Schoenfeld ER, Natarajan N (1990). Patterns of milk consumption and risk of cancer. Nutr Cancer.

[35] Park Y, Leitzmann MF, Subar AF, Hollenbeck A, Schatzkin A (2009). Dairy food, calcium, and risk of cancer in the NIH-AARP Diet and Health Study. Arch Intern Med.

[36] Galanis DJ, Kolonel LN, Lee J, Nomura A (1998). Intakes of selected foods and beverages and the incidence of gastric cancer among the Japanese residents of Hawaii: a prospective study. Int J Epidemiol.

[37] Hansson LE, Nyren O, Bergstrom R, Wolk A, Lindgren A, Baron J, Adami HO (1993). Diet and risk of gastric cancer. A population-based case-control study in Sweden. Int J Cancer.

[38] van der Pols JC, Bain C, Gunnell D, Smith GD, Frobisher C, Martin RM (2007). Childhood dairy intake and adult cancer risk: 65-y follow-up of the Boyd Orr cohort. Am J Clin Nutr.

[39] Boeing H, Frentzel-Beyme R, Berger M, Berndt V, Gores W, Korner M, Lohmeier R, Menarcher A, Mannl HF, Meinhardt M, Muller R, Ostermeier H, Paul F (1991). Case-control study on stomach cancer in Germany. Int J Cancer.

[40] Cornee J, Pobel D, Riboli E, Guyader M, Hemon B (1995). A case-control study of gastric cancer and nutritional factors in Marseille, France. Eur J Epidemiol.

[41] Lazarevic K, Nagorni A, Rancic N, Milutinovic S, Stosic L, Ilijev I (2010). Dietary factors and gastric cancer risk: hospital-based case control study. J Buon.

[42] Lissowska J, Gail MH, Pee D, Groves FD, Sobin LH, Nasierowska-Guttmejer A, Sygnowska E, Zatonski W, Blot WJ, Chow WH (2004). Diet and stomach cancer risk in Warsaw, Poland. Nutr Cancer.

[43] Lucenteforte E, Scita V, Bosetti C, Bertuccio P, Negri E, La Vecchia C (2008). Food groups and alcoholic beverages and the risk of stomach cancer: a case-control study in Italy. Nutr Cancer.

[44] Hoshiyama Y, Sasaba T (1992). A case-control study of stomach cancer and its relation to diet, cigarettes, and alcohol consumption in Saitama Prefecture, Japan. Cancer Causes Control.

[45] Huang XE, Tajima K, Hamajima N, Xiang J, Inoue M, Hirose K, Tominaga S, Takezaki T, Kuroishi T, Tokudome S (2000). Comparison of lifestyle and risk factors among Japanese with and without gastric cancer family history. Int J Cancer.

[46] Kim HJ, Chang WK, Kim MK, Lee SS, Choi BY (2002). Dietary factors and gastric cancer in Korea: a case-control study. Int J Cancer.

[47] Fei SJ, Xiao SD (2006). Diet and gastric cancer: a case-control study in Shanghai urban districts. Chin J Dig Dis.

[48] Yu GP, Hsieh CC (1991). Risk factors for stomach cancer: a population-based case-control study in Shanghai. Cancer Causes Control.

[49] Inoue M, Tajima K, Hirose K, Kuroishi T, Gao CM, Kitoh T (1994). Life-style and subsite of gastric cancer--joint effect of smoking and drinking habits. Int J Cancer.

[50] Gao Y, Hu N, Han XY, Ding T, Giffen C, Goldstein AM, Taylor PR (2011). Risk factors for esophageal and gastric cancers in Shanxi Province, China: a case-control study. Cancer Epidemiol.

[51] Pourfarzi F, Whelan A, Kaldor J, Malekzadeh R (2009). The role of diet and other environmental factors in the causation of gastric cancer in Iran--a population based study. Int J Cancer.

[52] Mathew A, Gangadharan P, Varghese C, Nair MK (2000). Diet and stomach cancer: a case-control study in South India. Eur J Cancer Prev.

[53] Ko KP, Park SK, Yang JJ, Ma SH, Gwack J, Shin A, Kim Y, Kang D, Chang SH, Shin HR (2013). Intake of soy products and other foods and gastric cancer risk: a prospective study. Journal of Epidemiology.

[54] Ito LS, Oba-Shinjo SM, Marie SK, Uno M, Shinjo SK, Hamajima N, Tajima K, Tominaga S (2003). Lifestyle factors associated with atrophic gastritis among Helicobacter pylori-seropositive Japanese-Brazilians in Sao Paulo. Int J Clin Oncol.

[55] Somi MH, Mousavi SM, Naghashi S, Faramarzi E, Jafarabadi MA, Ghojazade M, Majidi A, Naseri AS (2014). Is there any relationship between food habits in the last two decades and gastric cancer in North-Western Iran?. Asian Pacific journal of cancer prevention.

[56] Chen MJ, Wu DC, Lin JM, Wu MT, Sung FC (2009). Etiologic factors of gastric cardiac adenocarcinoma among men in Taiwan. World J Gastroenterol.

[57] Watabe K, Nishi M, Miyake H, Hirata K (1998). Lifestyle and gastric cancer: a case-control study. Oncol Rep.

[58] Munoz N, Plummer M, Vivas J, Moreno V, De Sanjose S, Lopez G, Oliver W (2001). A case-control study of gastric cancer in Venezuela. Int J Cancer.

[59] De Stefani E, Correa P, Boffetta P, Deneo-Pellegrini H, Ronco AL, Mendilaharsu M (2004). Dietary patterns and risk of gastric cancer: a case-control study in Uruguay. Gastric Cancer.

[60] Ward MH, Lopez-Carrillo L (1999). Dietary factors and the risk of gastric cancer in Mexico City. Am J Epidemiol.

[61] Icli F, Akbulut H, Yalcin B, Ozdemir F, Isikdogan A, Hayran M, Unsal D, Coskun S, Buyukcelik A, Yamac D (2010). Education, economic status and other risk factors in gastric cancer: “a case-control study of Turkish Oncology Group”. Med Oncol.

[62] Memik F, Nak SG, Gulten M, Ozturk M (1992). Gastric carcinoma in northwestern Turkey: epidemiologic characteristics. J Environ Pathol Toxicol Oncol.

[63] Outwater JL, Nicholson A, Barnard N (1997). Dairy products and breast cancer: the IGF-I, estrogen, and bGH hypothesis. Med Hypotheses.

[64] Matsubara J, Yamada Y, Hirashima Y, Takahari D, Okita NT, Kato K, Hamaguchi T, Shirao K, Shimada Y, Shimoda T (2008). Impact of insulin-like growth factor type 1 receptor, epidermal growth factor receptor, and HER2 expressions on outcomes of patients with gastric cancer. Clin Cancer Res.

[65] Alonso-Amelot ME, Castillo U, Smith BL, Lauren DR (1996). Bracken ptaquiloside in milk. Nature.

[66] Alonso-Amelot ME, Avendano M (2001). Possible association between gastric cancer and bracken fern in Venezuela: an epidemiologic study. Int J Cancer.

[67] Qin LQ, Wang PY, Kaneko T, Hoshi K, Sato A (2004). Estrogen: one of the risk factors in milk for prostate cancer. Med Hypotheses.

[68] Green J, Czanner G, Reeves G, Watson J, Wise L, Roddam A, Beral V (2011). Menopausal hormone therapy and risk of gastrointestinal cancer: nested case-control study within a prospective cohort, and meta-analysis. Int J Cancer.

[69] DerSimonian R, Laird N (1986). Meta-analysis in clinical trials. Control Clin Trials.

[70] Lau J, Ioannidis JP, Schmid CH (1997). Quantitative synthesis in systematic reviews. Ann Intern Med.

[71] Zintzaras E, Ioannidis JP (2005). Heterogeneity testing in meta-analysis of genome searches. Genet Epidemiol.

[72] Egger M, Davey Smith G, Schneider M, Minder C (1997). Bias in meta-analysis detected by a simple, graphical test. Bmj.

[73] Begg CB, Mazumdar M (1994). Operating characteristics of a rank correlation test for publication bias. Biometrics.

