# Supplementary material for: Milk/dairy products consumption and gastric cancer: an update meta-analysis of epidemiological studies
Source: Oncotarget. 2017 Dec 15;9(6):7126–35. doi: 10.18632/oncotarget.23496 (PMC5805541; doi:10.18632/oncotarget.23496)
Supplement: Supplementary file 1 [file oncotarget-09-7126-s001.pdf]

Milk/dairy products consumption and gastric cancer: an update meta-analysis of epidemiological studies

SUPPLEMENTARY MATERIALS

Supplementary Table 1: Characteristics of studies included in the meta-analysis. See Supplementary_Table_1
