# Supplementary material for: Milk/dairy products consumption and gastric cancer: an update meta-analysis of epidemiological studies
Source: Oncotarget. 2017 Dec 15;9(6):7126–35. doi: 10.18632/oncotarget.23496 (PMC5805541; doi:10.18632/oncotarget.23496)
Supplement: Supplementary file 2 [file oncotarget-09-7126-s002.docx]

Supplementary Table 1. Characteristics of studies included in the meta-analysis.

| Study, (Publication Year, Country) | Study design | Study period | Case/Control  （age yrs） | Items | Dairy products consumption levels | Exposure assessment  (Dietary exposure period) | Quality  assessment  score | Adjusted Variables |
| --- | --- | --- | --- | --- | --- | --- | --- | --- |
| Pourfarzi et al.,^51^  (2009 Iran) | PB case-control | 2004-2005 | 217/394  (> 18y) | Dairy products | ≥ 1 time/day  vs. ≤ 2 times/week | Face-to-face interviews  A structured questionnaire  (NS) | 7 | gender, age group, education, family history of GC, citrus fruits, garlic, onion, red meat, fish, strength and warmth of tea, preference for salt intake and H. pylori. |
| Lazarevic et al., ^41^  (2010 Serbia) | HB  case-control | 2005-2006 | 102/204  (most 50-80) | Milk  Dairy | Highest tertile  vs. Lowest tertile | FFQ  (1 year prior to diagnosis) | 7 | age, sex, residence, education, meals regularity, tobacco smoking, and history of cancer in first-degree relatives |
| Hansson et al., ^37^  (1993 Sweden) | PB  case-control | 1989-1992 | 338/669  (40-79y) | Whole milk  Cheese | ≥ 6900 ml/week  vs. ≤ 199 ml/week | Interview FFQ  (20 years prior to  interview ) | 6 | age, gender, SES |
| Kim et al., ^46^  (2002 Korea) | HB  case-control | 1997-1998 | 136/136  (55-74y) | Milk and milk products | Highest quarter  vs. Lowest quarter | Quantitative food  frequency method  (NS) | 6 | sex, age, SES, family history and refrigerator use |
| Munoz et al., ^58^  (2001 Venezuela) | PB  case-control | 1991-1997 | 302/485  (>35y) | Dairy products | Highest quarter vs. Lowest quarter | semi-quantitative FFQ  (NS) | 7 | age, sex, tobacco, alcohol, total calories and SES |
| Mathew et al.,^52^  (1999 India) | HB  case-control | 1988-1991 | 194/305  (≥ 20y) | Milk | > 1cup/day vs. never | FFQ  (NS) | 7 | age, sex |
| Correa et al., ^30^  (1985 USA) | HB  case-control | 1979-1983 | 391/391  (NS) | Dairy products | NS | Dietary questionnaire  (prior to the onset of the illness) | 7 | age, sex, respondent status, education, tobacco, alcohol use, income |
| Hoshiyama et al., ^44^  (1992 Japan) | PB  case-control | 1984-1990 | 294/294  (NS) | Dairy products | ≥ 5 times/week  vs. ≤ 1 time/week | Dietary questionnaire  (NS) | 7 | sex, age, smoking status, administrative division |
| Boeing et al., ^39^  (1991 Germany) | HB  case-control | 1985-1987 | 143/579  (32-80y) | Milk;  Dairy;  Cheese | Highest tertile  vs. Lowest tertile | Interviewer-administered questionnaire  (5 years before interview) | 6 | age, sex, and hospital |
| Palli et al., ^27^  (1992 Italy) | PB  case-control | 1985-1987 | 923/1159  (≤ 75y) | Milk/dairy products | Highest tertile  vs. Lowest tertile | structured questionnaire  (2 years before the interview) | 6 | age, sex |
| Chen et al., ^31^  (2002 USA) | PB  case-control | 1988-1993 | 124/449  (≥ 21 y) | Dairy products;  Milk | Highest quarter  vs. Lowest quarter | Questionnaire, modified version of the short Health Habits and History;  (recall their frequency of consumption before 1985) | 7 | age, sex, energy intake, BMI, respondent type, alcohol use, tobacco use, education, family history, vitamin supplement |
| Wu-Williams et al., ^33^ (1990 USA) | PB  case-control | 1975-1982 | M 137/137  (<55y) | Milk | ≥ 5 times/week  vs. ≤ 1 time/week | structured questionnaire  (NS) | 5 | age, sex, race |
| Huang et al., ^45^  (2000 Japan) | HB  case-control | 1988-1995 | 1111/26996  (40-79y) | Milk | 1 time/day  vs. sometimes | Self-administered questionnaires  (Before symptoms appeared) | 5 | age, sex |
| Cornee et al., ^40^  (1995 France) | HB  case-control | 1985-1988 | 92/128  (Case: 66.6y;  Control: 66.5y) | Dairy;  Milk | Highest tertile  vs. Lowest tertile | dietary history questionnaire developed by INSERM  (the year preceding the interview or symptoms) | 6 | age, sex, occupation, energy intake |
| Ward et al., ^60^  (1999 Mexico) | PB  case-control | 1989-1990 | 220/752  (≥ 20y) | Dairy;  Milk;  Cheese; | ≥ 1 time/day  vs. < 1 time/week | Semiquantitative food frequency  (1 year before onset of symptoms or interview) | 8 | age, gender, calories, chili pepper intake, cigarette smoking, SES, added salt, and history of pepticulcer disease |
| Yu et al., ^48^  (1991 China) | PB  case-control | 1976-1980 | 84/2676  (NS) | Milk | Users vs. Nonusers | standardized questionnaire  (NS) | 8 | age, sex, family income, fruit, family history of stomach cancer, family history of other cancer, history of tuberculosis, blood type, cigarette smoking, alcohol, strong tea |
| De Stefani et al., ^59^  (2004 Uruguay) | HB  case-control | 1996-2000 | 249/960  (30-89y) | Dairy foods | Highest tertile  vs. Lowest tertile | FFQ  (5 years before the first symptom) | 7 | age, sex, residence, urban/ rural status, education, BMI, and total energy intake |
| Inoue et al., ^49^  (1994 Japan) | HB  case-control | 1988-1991 | 668/668  (Case: 58.0y;  Control: 57.8y) | Milk | ≥ 3-4 times/week  vs. less | self-administered questionnaire sheets  (before the present illness became apparent) | 6 | age, sex |
| Lissowska et al., ^42^  (2004 Poland) | PB  case-control | 1994-1996 | 274/463  ( most ≥ 50y) | Dairy products | Highest quarter vs. Lowest quarter | a modification of the Block questionnaire FFQ  (prior to 1990) | 7 | age, sex, education, smoking, and calories from food |
| Fei et al., ^47^  (2006 China) | HB  case-control | 2004-2006 | 189/567  (28-93y) | Milk products | Users vs. Nonusers | questionnaire  (NS) | 5 | age, sex |
| Mettlin et al., ^34^  (1990 USA) | HB  case-control |  | 115/1300  (NS) | Whole milk;  Skim milk | Daily vs. None | standardized admissions questionnaire  (NS) | 6 | sex, age, county of residence, smoking history, education |
| Icli et al., ^61^  (2010 Turkey) | PB  case-control | 2005 | 253/253  (Case: 55.5y;  Control: 57.0y) | Milk | High frequency vs. Low frequency | Interviewed questionnaire  (period before their  gastric disease) | 5 | age, gender, the city of residence, the level of education |
| Lucenteforte et al.,^43^  (2008 Italy) | HB  case-control | 1997-2007 | 230/547  (22-80y) | Milk and yoghurt | Highest quintile  vs. Lowest quintile | Interviewed  FFQ  (2 yr before diagnosis or hospital admission) | 7 | sex, age, education, year of interview, BMI, tobacco smoking, family history of stomach cancer, total energy intake |
| Gao et al., ^50^  (2011 China) | PB  case-control | 1997-2005 | 915/915  (61y) | Milk/dairy products | Ever vs. Never | interviewer-administered questionnaire  (before ill or interview) | 8 | age, gender, geographic  region(5 classes); |
| Memik et al., ^62^  (1992 Turkey) | HB  case-control | 1977-1991 | 252/609 (case: M 58y, F 56y; Control: 60y) | Milk | 600+ml/week vs. 0-200ml/week | NS  (NS) | 5 | age, sex |
| Ito et al^54^., (2003 Japan) | HB  case-control | 1988-1998. | F 508/36490  (≥30y) | Milk | Highest quarter vs. Lowest quarter | FFQ checked by a trained interviewer  (NS) | 7 | Age, year and season of fist hospital visit, smoking habit, family history of gastric cancer |
| Chen et al^56^., (2009 China) | HB  case-control | 2000-2009 | M 41/205  (Case: 64.5y;  Control: 63.3y) | Dairy products  Milk | ≥3 time/week  vs. <3 time/week  yes vs. no | FFQ (≥1 yr before diagnosis or interviewed) | 6 | Age and years of schooling |
| Somi et al.,^55^  ( 2015 Iran) | HB  case-control | 2009-2011 | 212/404  ( 21-84y) | Milk  Cheese | yes vs. no | FFQ (over the past two decades) | 8 | sex, age, educational  level and history of smoking |
| Watabe et al.,^57^  (1998 Japan) | PB  case-control | 1996-1997 | 242/484  (40-79y) | Milk  Cheese | Daily vs. not  ≥3 time/week  vs. <3 time/week | FFQ (10 years ago) | 5 | Age, sex, place of resident |
| Nomura et al.,^32^  (1990 USA) | Cohort | 1965-1986  follow-up 19 y | M 7990  (Born between 1900 and 1919) | Milk; Cheese;  Ice cream | ≥ 5 time/week  vs. ≤ 1time/week | FFQ: based on a 24h diet recall history  (NS) | 8 | age |
| van der Pols et al., ^38^  (2007 UK) | Cohort | 1948-2005 follow-up 65 y | 4999  (4–11y) | Dairy products; Milk | 282 ml/day  vs. < 118 ml/day | A 7-d household inventory method  childhood dairy consumption | 7 | age, sex, energy, fruit, calcium intakes |
| Park et al., ^35^  (2009 USA) | Cohort | 1995-2003  follow-up  7 y | 293907M+198903F  (50-71y) | Dairy foods | Highest quintile  vs. Lowest quintile | FFQ | 8 | smoking, and antacid use |
| Galanis et al.^36^ (1998 USA) | Cohort | 1975-1994  follow-up 14.8y | 6297F+5610M  (≥ 18y) | Milk | ≥ 1 cups/day vs. none | A short questionnaire  (NS) | 9 | Age, years of education,  Japanese place of birth, gender. Among man also adjusted for cigarette smoking and alcohol intake status |
| Ko et al.,^53^  (2013 South Korea) | Cohort | 1993-2004  follow-up 8.5y | 9724( 30-90y) | Dairy products | ≥ 1 time/d vs. almost never | a brief 14-FFQ  (NS) | 9 | age, sex, cigarette smoking, BMI, alcohol drinking, area of residence |

PB: population-based; HB: hospital-based; M: male; F: female; NS: not specified; GC: gastric cancer; SES: Socio-economic status; BMI: body mass index; FFQ: food frequency questionnaire ; INSERM : the French National Institute of Health and Medical Research
